# Soil class map of the Rio Jardim watershed in Central Brazil at 30 meter spatial resolution based on proximal and remote sensed data and MESMA method

**DOI:** 10.1016/j.dib.2019.104070

**Published:** 2019-05-30

**Authors:** Raúl R. Poppiel, Marilusa P.C. Lacerda, José A.M. Demattê, Manuel P. Oliveira, Bruna C. Gallo, José L. Safanelli

**Affiliations:** a Faculty of Agronomy and Veterinary Medicine, University of Brasília; ICC Sul, Darcy Ribeiro University Campus, Asa Norte, Postal Box 4508, Brasília 70910-960, Brazil; b Department of Soil Science, College of Agriculture Luiz de Queiroz, University of São Paulo; Pádua Dias Av., 11, Piracicaba, Postal Box 09, São Paulo 13416-900, Brazil

**Keywords:** Digital soil mapping, Soil management, Agricultural planning, Soil classification system, Landsat

## Abstract

Geospatial soil information is critical for agricultural policy formulation and decision making, land-use suitability analysis, sustainable soil management, environmental assessment, and other research topics that are of vital importance to agriculture and economy. Proximal and Remote sensing technologies enables us to collect, process, and analyze spectral data and to retrieve, synthesize, visualize valuable geospatial information for multidisciplinary uses. We obtained the soil class map provided in this article by processing and analyzing proximal and remote sensed data from soil samples collected in toposequences based on pedomorphogeological relashionships. The soils were classified up to the second categorical level (suborder) of the Brazilian Soil Classification System (SiBCS), as well as in the World Reference Base (WRB) and United States Soil Taxonomy (ST) systems. The raster map has 30 m resolution and its accuracy is 73% (Kappa coefficient of 0.73). The soil legend represents a soil class followed by its topsoil color.

**Table:** Specifications table

Subject area	Environmental Earth and Soil Sciences
More specific subject area	Pedology, Soil Geography, Soil Management
Type of data	Digital soil map (raster kmz file), movie (3D animation mp4 file)
How data was acquired	Soils were sampled according to pedomorphogeological relationships and were determined morphological and physico-chemical attributes. Topsoil reflectance was analyzed in laboratory using the FieldSpec Pro sensor (Analytical Spectral Devices Inc., Boulder, CO, USA). We acquired 10 Landsat 5 TM images corresponding to orbit 221 and point 71 between May and September (dry season) from 1984 to 2009.
Data format	Processed
Experimental factors	Soils were classified up field up to the second categorical level (suborder), according to the Brazilian Soil Classification System – SiBCS, and topsoil patterns were determined to support soil class mapping by applying the Multiple Endmember Spectral Mixture Analysis (MESMA) to a bare soil composite (image).
Experimental features	The detailed digital mapping product (30 m) provides soil class information at suborder level in the WRB, SiBCS and ST classification systems, performed for an area of 53,614 ha in Central Brazil, from proximal and remoted sensed data by MESMA method. The map accuracy is 73% (Kappa coefficient of 0.73), indicating very good agreement between the map and the verification sites.
Data source location	The area is located in the Rio Jardim watershed, southeast of Federal District (DF), Brazil, from 216,6 to 226,9 m E, and 8,261,507 to 8,244,566 m N, UTM projection, fuse 23 South, SIRGAS 2000 datum.
Data accessibility	Data are available within the article.
Related research article	Poppiel, R.R.; Lacerda, P.C.L.; Demattê, J.A.M.; Oliveira Jr, M.P.; Gallo, B.C.; Safanelli, J.L. Pedology and soil class mapping from proximal and remote sensed data. Geoderma, 2019 (in press).

**Table:** 

**Value of the data** • The soil data of the map is up to date with pedologic knowledge and current demands. • The soil class map can be used to evaluate the locations most suitable for agriculture and expansion of urban/settlement areas and industry; to guide sustainable soil management and planning; to identify areas for protection or conservation; to indicate areas with greater risk of soil degradation; to optimize sampling in precision agriculture; to support irrigation projects, land pricing and purchase, etc. • Detailed soil map can be implemented from small-farm to basin scale for making land use decisions. • Map available here can be served as guideline for other studies highlighting in the field of hydrological modelling, experimental design in agronomy, forestry and biology to define the study area and to allocate the factors. As well can be used as reference for validation purpose of other studies on digital soil mapping.

## 1 Data

The dataset contains spatial variability of soil classes at detailed scale (30 m spatial resolution), classified according to the Brazilian Soil Classification System – SiBCS [1], the World Reference Base – WRB [2] and the United States Soil Taxonomy – ST [3].

**Fig. 1:**
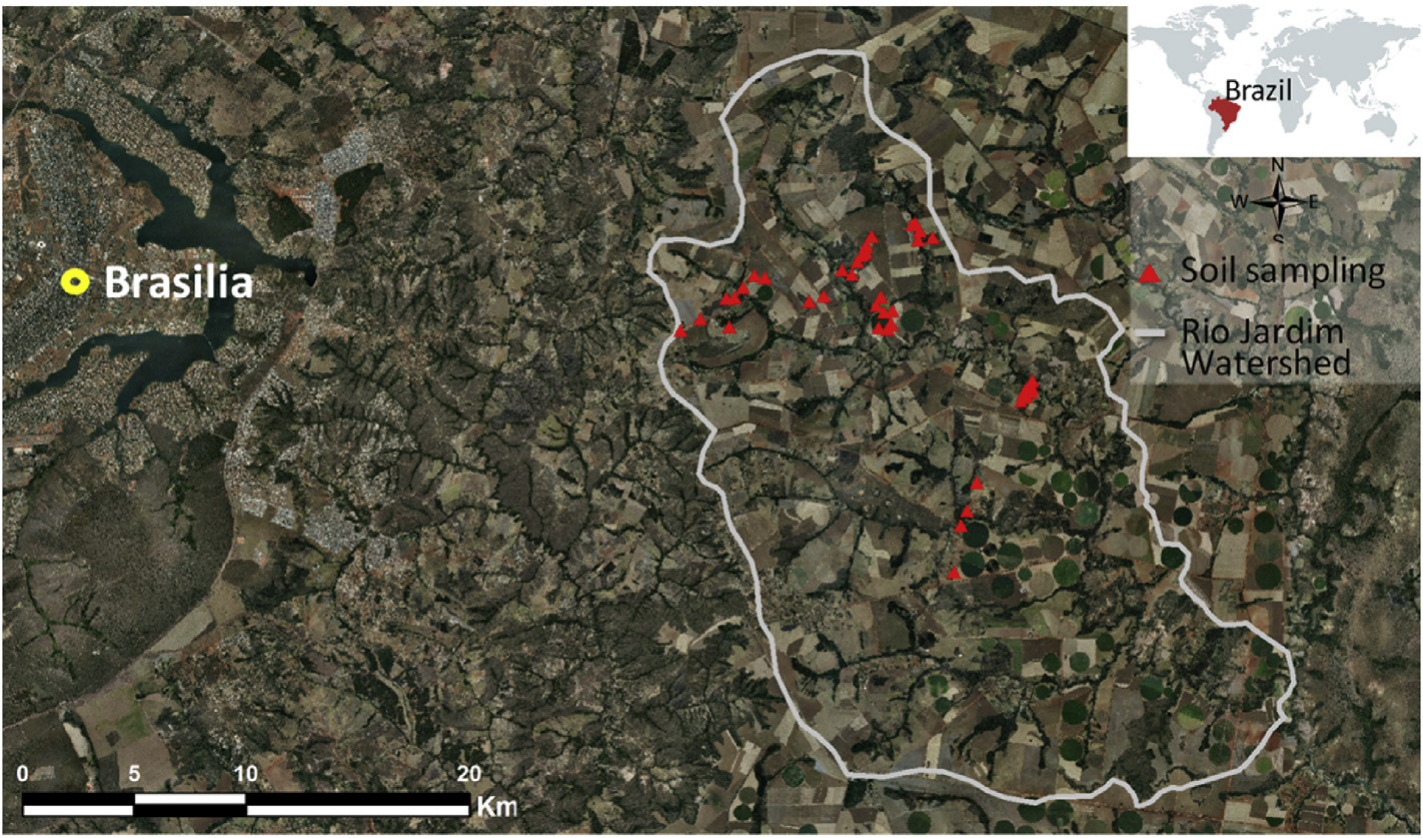
Dataset location, soil sampling points and aerial photography with natural color composite.

**Fig. 2:**
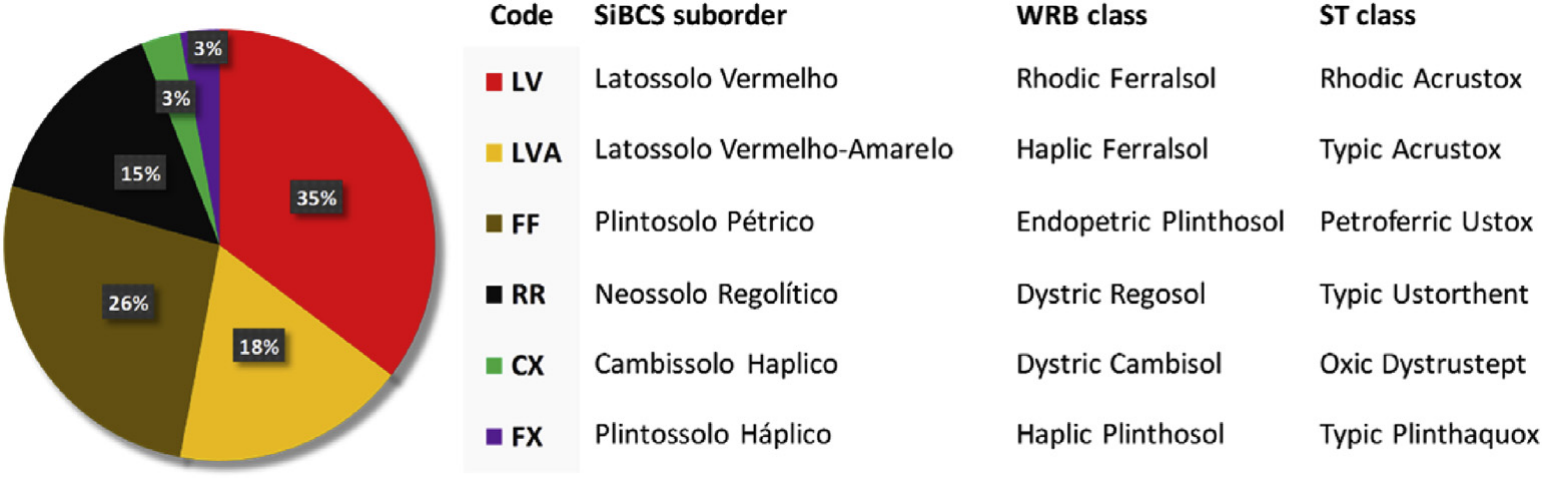
Soil observations according to the Brazilian Soil Classification System – SiBCS [1] and corresponding World Reference Base – WRB [2] and Soil Taxonomy – ST [3] classifications.

**Fig. 3:**
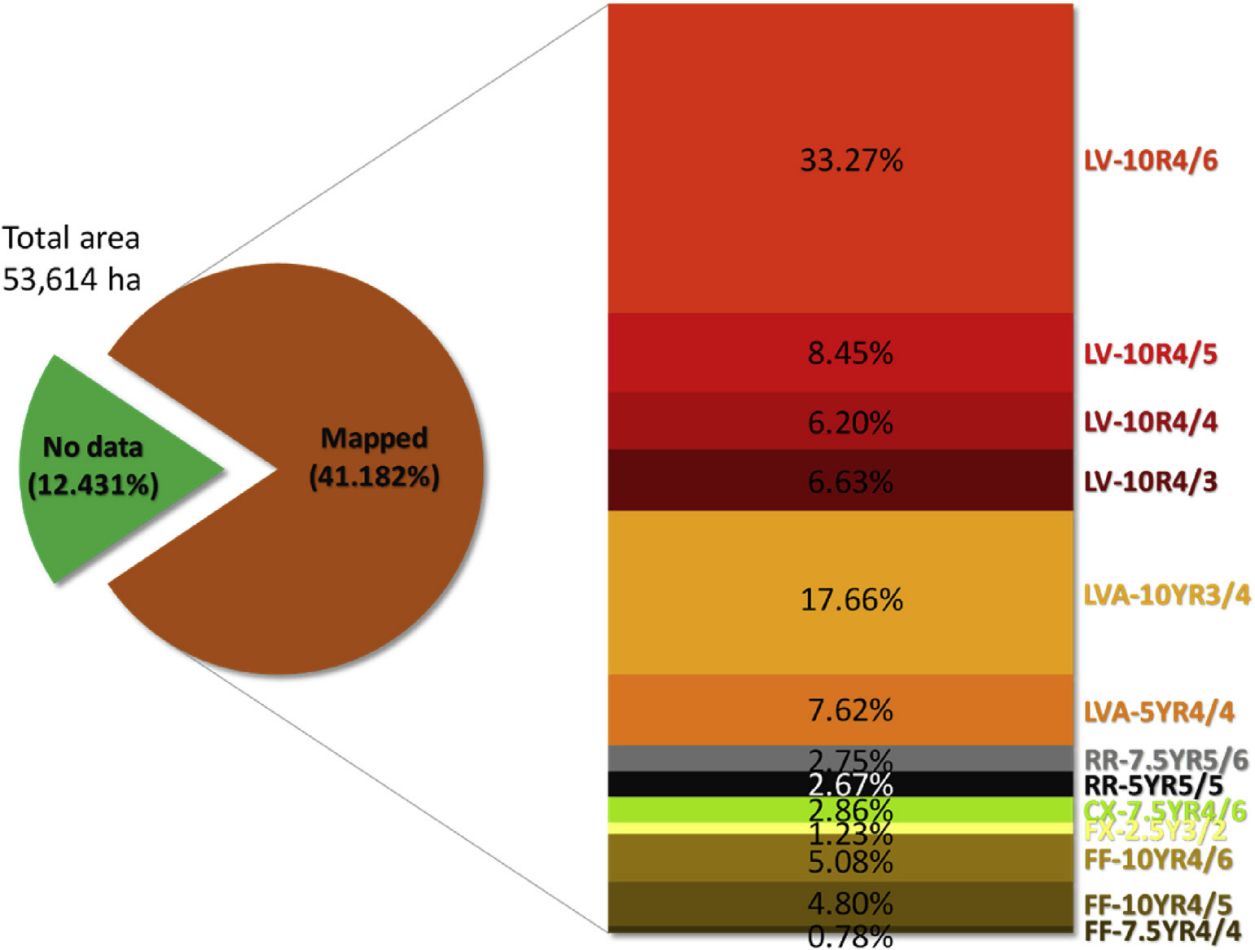
Summary quantifications of the soil map. LV: Rhodic Ferralsol; LVA: Haplic Ferralsol; RR: Dystric Regosol; CX: Dystric Cambisol; FX: Haplic Plinthosol; FF: Endopetric Plinthosol.

The sampling points were distributed over the Rio Jardim Watershed, in Central Brazil (Fig. 1), where we collected soil samples of Ferralsols, Plinthosols, Regosols and Cambisols (Fig. 2). After analyzing and processing all the data, we mapped the soils and quantified the results (Fig. 3). The soil map was saved in a KMZ file and provided in a 3D movie overlapped on the digital elevation model (Supplementary materials).

## 2 Experimental design, materials and methods

### 2.1 Soil sampling

The soil sampling design based on the soil-geoform-geology (pedomorphogeological) relationships allowed to identify representative areas where we defined six toposequences and 34 sites to visit in the field. In each site, we performed morphological description according to Santos et al. [4], and we collected soil samples from surface A horizon and diagnostic subsurface horizons (B or C), as well as at 0–0.2, 0.2–0.4 and 0.8–1.0 m layers. The soils were classified up to the second categorical level (suborder), according to the Brazilian Soil Classification System – SiBCS [1] (Fig. 2). For the validation step, we used an external and independent dataset of 231 sites distributed on a regular grid of 1400 × 1400 m.

### 2.2 Soil attributes analysis

The soil samples were air-dried, ground and sieved (2 mm mesh) and analyzed for physical and chemical determination according to Embrapa [5]. The spectroscopic analysis was conducted using the FieldSpec Pro sensor (Analytical Spectral Devices Inc., Boulder, CO, USA), which has 1 nm of spectral resolution from 350 nm to 2500 nm (VIS-NIR-SWIR).

### 2.3 Satellite images acquisition

We acquire a Landsat 5 TM Level-1 time series (VIS-NIR-SWIR range) with 10 images from the United States Geological Survey (USGS), corresponding to orbit 221 and point 71 between May and September (dry season) from 1984 to 2009, with up to 10% maximum cloud cover.

### 2.4 Data processing

Laboratory topsoil reflectance spectra were processed by removing the continuous spectrum (CR) [6] and by applying the second derivative (SD) of the Kubelka–Munk (K-M) function [7] from 350 to 2500nm for mineralogical assessment. We clustered soils with similar attributes based on topsoil reflectance to obtain spectral patterns, which were convolved using a Gaussian function of Landsat 5 TM spectral bands to obtain endmembers. Then, we added to each cluster (a soil class) the main topsoil attribute for creating soil legend for the map. Poppiel et al. [8] described further information about the method.

The raw digital numbers in the images were converted to radiance and then to reflectance-at-surface values [9] by applying the module Fast Line-of-sight Atmospheric Analysis of Hyperspectral cubes (FLAASH) using the ENVI software. We obtained bare soil images by applying the Normalized Difference Vegetation Index – NDVI [10] to suppress vegetated areas (dense, moderate and sparse), and the Middle Infrared Index-MIDII [11] to mask areas with straw or burned. Then, the bare soil reflectance was retrieved in a bare soil composite (single image), denominated Synthetic Soil Image (SYSI) by selecting the lowest value of the MIDII which is correlated with low moisture content using the R programing language [12], according to [8]. We applied the Multiple Endmember Spectral Mixture Analysis (MESMA) [13] to address the inter-class and intra-class soil spectral variability using as inputs data the SYSI and the endmembers to select the mixture model (a soil class) that best fits each pixel. To improve spatial coherence, we smoothed the resulting image by applying a median filter, using a 7 × 7 kernel.

For assessment of the map accuracy, we calculated the Kappa coefficient [14] based on a confusion matrix, and its values were classified as proposed by [15]. Poppiel et al. [8] presented a flowchart to illustrate the complete methodology. We showed summary quantifications of the mapped soil classes in Fig. 3.
